# Occurrence and molecular characterization of *Meloidogyne graminicola* on rice in Central Punjab, Pakistan

**DOI:** 10.21307/jofnem-2020-123

**Published:** 2021-01-16

**Authors:** Abdul Jabbar, Nazir Javed, Anjum Munir, Huma Abbas, Sajid A. Khan, Anam Moosa, Muhammad Jabran, Byron J. Adams, Muhammad A. Ali

**Affiliations:** 1 Department of Plant Pathology, University of Agriculture Faisalabad, P.O. Box 38040, Pakistan; 2 Crop Diseases Research Institute, NARC, Islamabad, Pakistan; 3 Department of Biology, Monte L. Bean Museum, and Evolutionary Ecology Laboratories, Brigham Young University, Provo, UT 84602

**Keywords:** Alternate hosts, Diagnosis, ITS rDNA, *Meloidogyne graminicola*, Morphology, Rice root-knot nematode

## Abstract

*Meloidogyne graminicola* threatens global rice production, yet is understudied for many areas where it is cultivated. To better understand the prevalence and incidence of *M. graminicola* in central Punjab, Pakistan, we carried out field surveys of rice fields in the districts of Faisalabad and Chiniot. *M. graminicola* isolates were recovered from soil and root samples and identified on the basis of perineal patterns and rDNA ITS-based sequencing. The severity of nematode attack on rice roots and infested fields at various locations was based on galling index, root-knot nematode juveniles per root system, juveniles per 100 ml of soil, and prevalence of stylet-bearing nematodes and non-stylet-bearing nematodes. Maximum prevalence (22.5 and 27.5%) and minimum prevalence (17.5 and 20%) of *M. graminicola* was observed in Chiniot and Faisalabad, respectively. Eleven alternate host-plant species were examined in this study revealing varying degrees of *M. graminicola* infestation. ITS sequencing and phylogenetic analysis indicated that isolates from this study form a well-resolved clade with others from Asia, while another isolate falls outside of this clade in an unresolved polytomy with those from Europe and South America. Though monophyletic with the other *M. graminicola*, the isolates from Pakistan are distinguished by their high genetic variability and long branch lengths relative to the other isolates of *M. graminicola*, suggesting Pakistan as a possible ancestral area. Our results indicate that rice is severely attacked by a genetically diverse and aggressive *M. graminicola*, necessitating the development of appropriate control measures for its management in rice and other graminaceous crops.

Rice (*Oryza sativa* L.) is one of the major cereal crops produced in Pakistan and is cultivated on an area of 2,900,600 hectares with a production of 11,174,700 tons (FAO, 2017). As a staple food, its consumption exceeds 100 kg per capita annually in most of the Asian countries (Seck et al., 2012). Several biotic and abiotic constraints limit the yield and quality of rice. Among biotic constraints, plant-parasitic nematodes are an emerging threat to rice production. Root-knot nematodes (RKNs) are the most destructive plant-parasitic nematodes in upland, lowland, and deep-water rice cultivation systems (Panwar and Rao, 1998; Bridge et al., 2005). RKNs possess the ability to penetrate the roots, induce root galling, suppress plant defense mechanisms, hijack the plant’s metabolic system, and establish giant cells for the sake of their own benefit (Kyndt et al., 2014; Ali et al., 2017a, 2017b, 2018). As an outcome, plants gradually lose vigor, ultimately leading to substantial yield loss (Bridge et al., 2005; Win et al., 2015; Ali et al., 2015). Among various RKNs, *Meloidogyne graminicola* (Golden and Birchfield) has emerged as the most serious pest of rice (Mantelin et al., 2017). *M. graminicola* was first reported in Pakistan by Munir and Bridge (2003) during a survey of rice fields of Sheikhupura, Punjab, Pakistan. The subtropical climate of Pakistan and warm sandy soils are favorable for the development and reproduction of RKNs (Khattak, 2008). But plant-parasitic nematodes in these regions have received little attention in the past, and only a few surveys have been conducted to estimate their prevalence and incidence (Kafi, 1963; Ahmad and Khan, 1973; Saeed and Ashrafi, 1973; Khan et al., 2005; Zarina and Shahina, 2010; Anwar and McKenry, 2012). All varieties of rice grown in South Asian and Southeast Asian countries, whether upland or lowland, have exhibited susceptibility to *M. graminicola* (Padgham et al., 2004; Das et al., 2011; Win et al., 2013). Currently, there is little information about the association of *M. graminicola* with rice in Pakistan.

Compared with other phytopathogens, nematodes are difficult to control because they attack belowground parts of plants, resulting in reduced growth and yield loss (Williamson and Hussey, 1996). Moreover, nematodes are polyphagous pests that attack over 5,500 plant species, including several economically important crops (Moens and Perry, 2009; Ali et al., 2015; Ali et al., 2019). Successful control of *Meloidogyne* species can only be achieved by rapid and accurate identification of the nematode. Traditional techniques of nematode identification based on morphological features (Eisenback, 1985) are challenging because they are laborious and require extensive training and expertise. Historically, RKNs have been identified based primarily on morphological features. Currently, there is no information available about the molecular identification of *Meloidogyne* spp. in Pakistan, especially in regard to *M. graminicola*.

Several molecular techniques are available for the identification of *Meloidogyne* species (Blok and Powers, 2009). Among these techniques, the polymerase chain reaction (PCR) is a sensitive, quick, and accurate tool (Niu et al., 2011). Adam et al. (2007) developed a molecular diagnostic key that uses several molecular approaches to identify seven economically important RKNs that are frequently encountered in diagnostic laboratories. Moreover, with the increase in DNA-based sequencing, the tandem repeat unit segments of the 18 S, ITS1, 5.8 S, ITS2, and 28 S regions of the ribosomal DNA array (rDNA) and mitochondrial DNA (mtDNA) have proved to be efficient diagnostic tools for accurately identifying of RKNs (Landa et al., 2008; Naz et al., 2012).

Accurate identification of *M. graminicola*, as well as its prevalence and distribution spectra, is fundamental for applying management strategies in the field. Therefore, we used morphological and molecular approaches to identify RKNs in order to determine the distribution, prevalence, and disease intensity of *M. graminicola* in Chiniot and Faisalabad districts of Central Punjab, Pakistan.

## Materials and Methods

### Survey and sampling

A survey of rice-growing areas of Faisalabad and Chiniot districts of central Punjab, Pakistan, was conducted during September 2014, 2015, and 2016. Five root samples of rice plants and 1000-ml composite soil samples were collected from each sampling site, preserved in polythene bags, and labeled. The samples were transported to the Nematology Lab, Department of Plant Pathology, University of Agriculture, Faisalabad, for further processing. The roots were gently washed with distilled water to remove adhering soil and plant debris. Nematode prevalence was determined by using the formula of Norton (1978):

$$\mathrm{Nematode}\;\mathrm{prevalence}\;(\%)=\frac{\mathrm{Number}\;\mathrm{of}\;\mathrm{locations}\;\mathrm{infected}\;\mathrm{with}\;\mathrm{RKNs}}{\mathrm{Total}\;\mathrm{number}\;\mathrm{of}\;\mathrm{locations}\;\mathrm{surveyed}}\times 100$$

### Isolation and morphological identification of *M. graminicola*

Second-stage juveniles (J2s) of *M. graminicola* were isolated from rice roots. The root samples from each field were pooled, chopped into pieces, and mixed thoroughly. Three subsamples of 5-g roots were taken, and juveniles were isolated from infected roots using the Baermann funnel method and collected in a beaker. Nematode extraction from soil samples was carried out using Whitehead’s tray method (Whitehead and Hemming, 1965). Soil samples were mixed thoroughly, and a 100-ml composite sample was placed in a plastic bucket and 1–2 liters of water were added. Plant debris, heavy soil particles, and rocks were drained manually. The supernatant was sieved through a 50-mesh-size sieve in a separate bucket. The procedure was repeated by adding 1,000-ml water again and agitated properly. After that, the suspension was left to settle and poured again into another basket using a 100-mesh sieve. The process was repeated twice again using 250- and 325-mesh sieves. The final suspension was transferred to a 500-ml beaker and the supernatant was allowed to settle for one hour. Three subsamples of 2 ml each were taken and examined in a counting dish. Second-stage juveniles (J2) were counted using a stereomicroscope (Olympus SZ2-ILGB). Mature females of *M. graminicola* were identified based on micrographs of the internal and external perineal patterns (Jepson, 1987). Stained hooked gall tissues were teased apart with a pointed needle to separate mature females. The neck region of individual nematodes was excised, and the posterior part was dipped in 45% lactic acid solution to remove body tissues. The perineal patterns were trimmed and transferred on a transparent glass slide in a glycerin drop. External views of the perineal patterns were visualized using scanning electron microscopy (SEM) at Brigham Young University, Provo Utah, USA. Accordingly, specimens were dehydrated via ascending ethanol series prior to critical point drying. The animals were then mounted on stubs and gold-sputtered. Micrographs were recorded via Helios Nanolab 600 SEM (Thermo-Fisher Scientific, Hillsborough, USA).

### Statistical analysis

Data were subjected to statistical analysis using Statistix (Ver. 8.1). The experimental design for the analysis of galling index, juveniles/soil sample, juveniles/root sample, stylet, and non-stylet-bearing nematodes was by a completely randomized block design with treatment means separated using a LSD test at *P* = 0.05.

### DNA extraction

Isolates derived from single egg masses were used to extract DNA. Twenty larvae were picked, rinsed thrice with sterile distilled water, transferred to a microcentrifuge tube, and crushed in 500 µL of SDS extraction buffer containing Tris–HCl (1 M, pH 7.5), EDTA (0.5 M, pH 8.0), SDS (10% w/v), and dd H_2_O with a grinding plastic stick (Mitkowski et al., 2003). Proteins in the solution were digested by adding 20 µL proteinase K, 100 µg ml^−1^. Later, 500 µL of phenol (25:24:1, phenol/chloroform/isoamyl alcohol) was added and vortexed briefly. The tube was centrifuged at 14,000 rpm for 5 min and the supernatant was collected and transferred to another sterile microcentrifuge tube. Sodium acetate (3 M), 50 µL and ice-cold isopropanol, 500 µL, were added to the supernatant, vortexed gently, and centrifuged at 14,000 rpm for 5 min. The supernatant was discarded to save the pellet. Five-hundred µL of ethanol (96%) was added to the pellet and centrifuged for 3 min at 13,000 rpm. The supernatant was discarded again, and the pellet air-dried in a laminar flow chamber for 2 hr. DNA was dissolved in 30 µL of TE buffer (1 ml of 1 M Tris base (pH 8.0) and 0.2 ml of EDTA (0.5 M), dd H_2_O) and stored at –20°C until required for PCR reactions.

### Internal transcribed spacer region (ITS) amplification

The PCR reaction mixture was prepared by using 10 µL of 10X PCR buffer, 2-µL dNTPs (10 mM), 2-µL forward primer (10 µM), 2-µL reverse primer (10 µM), 2-µL template DNA (>  50 ng), 1-µL Taq polymerase, and 81-µL ddH_2_O, for a total reaction volume of 100 µL. PCR reactions were carried out in a thermal cycler (BIO-RAD T100^TM^) under the following conditions: (1) predenaturation at 94 °C for 5 min, (2) denaturation at 94°C for 1 min, (3) annealing at 64°C for 1 min, (4) extension at 72°C for 1 min, (5) 35 cycles of this process, and (6) final extension at 72°C for 5 min. The sequences of primers used for amplification of the internal transcribed spacer region are 1) forward primer rDNA2 (5’–TTGATTACGTCCCTGCCCTTT–3’) (Vrain et al., 1992) and reverse primer rDNA1. 5.8 s (5’–ACGAGCCCGAGTGATCCACCG–3’) (Cherry et al., 1997). Primers were synthesized by Sigma Genosys Inc, St. Louis, MO, USA.

### Gel electrophoresis and sequencing

The amplified PCR product was viewed on a gel stained with ethidium bromide under a UV transilluminator. DNA was excised from the gel and purified for sequencing using quick Gel Extraction Kit (Qiagen Gel Extraction Kit, Qiagen, Hilden, Germany). Purified DNA was sent for sequencing to L.G.C. Genomics, Germany, or the DNA sequencing center at Brigham Young University, Provo, Utah, USA.

### Phylogenetic analysis

Due to the high degree of interspecific variation in nucleotide sequences of nematodes (Hu and Gasser, 2006), the deduced internal transcribed spacer region sequences were used for phylogenetic study. The ITS sequences of the eight isolates were used to query the most similar curated sequences on GenBank by performing open-nucleotide BLAST (Basic Local Alignment Search Tool, Camacho et al., 2009). The nearest matches as well as those from closely related species of *Meloidogyne* (McClure et al., 2012) were downloaded from GenBank and used for subsequent phylogenetic analysis. Multiple-sequence alignment was generated using MUSCLE (Edgar, 2004). The alignment settings included optimization of profile-dependent parameters. Sequences were first grouped by similarity with anchor optimization. Iteration 1  =  kmer4_6 with pctid_kimura for subsequent interactions using the UPMGB clustering method and pseudo-tree rooting. CLUSTALW was used for the distance weighting scheme, with 32-base anchor spacing and 24-base minimum diagonals. Bayesian analyses were carried out using MrBayes 3.2.6 (Huelsenbeck and Ronquist, 2001). A general time-reversible model of sequence evolution with four categories of gamma rate variation was chosen as the optimal model of sequence evolution as per Posada and Crandall (1998). Bayesian analysis was initiated with a random starting tree with four heated chains of chain length 1,100,000, burn-in length of 100,000, and subsampling frequency of 200. Branch lengths were unconstrained.

## Results

### Prevalence of *M. graminicola*

**Figure 1:**
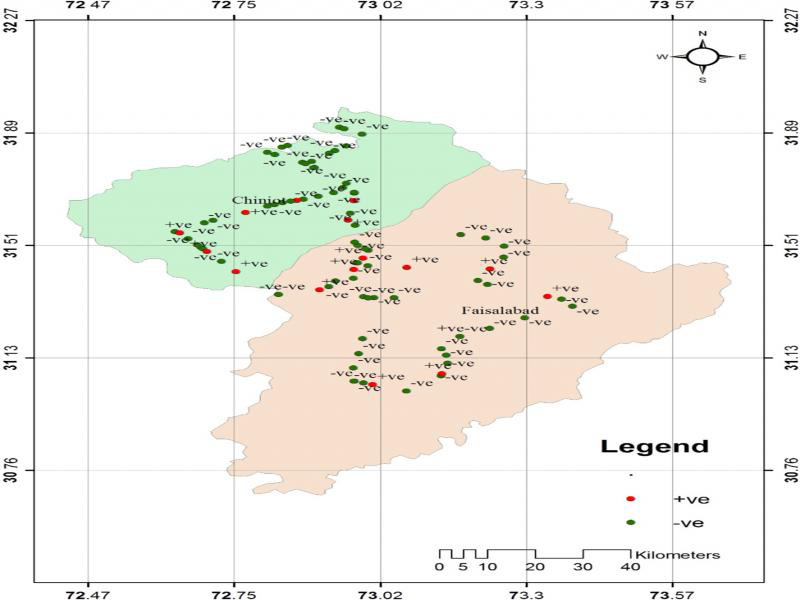
GPS map of Faisalabad and Chiniot districts. Red dots indicate locations of samples infested with *M. graminicola*.

**Figure 2:**
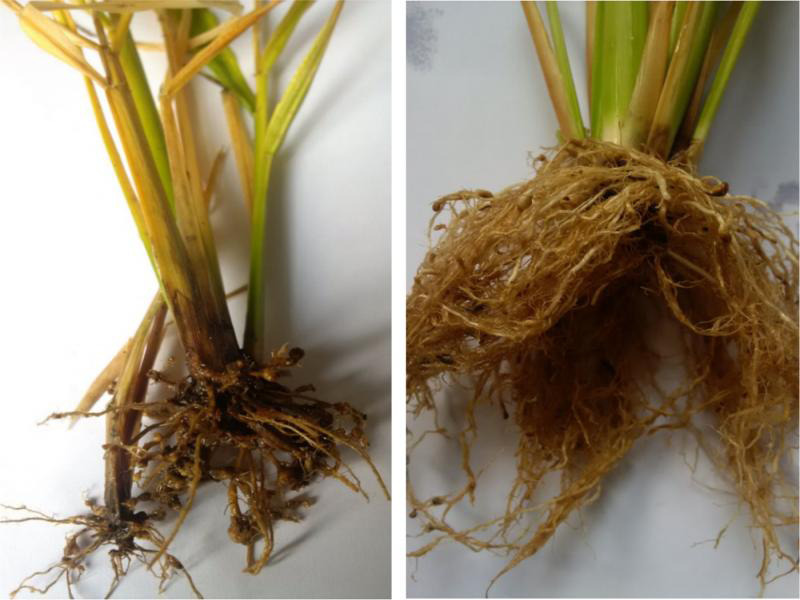
Rice roots infected with *M. graminicola*. The roots are showing typical symptoms of galls or knots.

The field survey data during the rice cropping season of 2014, 2015, and 2016 indicated that both districts Chiniot and Faisalabad have *M. graminicola* infestation during all the seasons. The GPS coordinates for each sample were used to plot the presence and absence of *M. graminicola* at the sampled sites in Faisalabad and Chiniot (Figure 1). The prevalence of *M. graminicola* at forty surveyed locations of Chiniot and Faisalabad is given in Tables 1 and 2. Maximum prevalence of *M. graminicola* (22.5 and 27.5%) was observed in Chiniot and Faisalabad, respectively, during the rice-growing season of 2016, while the minimum prevalence (17.5 and 20%, respectively) was recorded in Chiniot and Faisalabad during the cropping season of 2014. During 2014 to 2016 at Chiniot among 40 surveyed locations, the presence of *M. graminicola* was observed at 7, 8, and 9 locations respectively. Similarly, 40 locations were surveyed in Faisalabad during 2014–2016, and *M. graminicola* prevalence was recorded at 8, 10, and 11 locations, respectively. *M. graminicola* showed typical hook-shaped galls on rice roots that are a characteristic feature of *M. graminicola* infection (Figure 2).

**Table 1.** Prevalence of *M. graminicola* in Chiniot district.

					Prevalence		
Sr #	Locations	Elevation	Latitude	Longitude	2014	2015	2016
1	133 J.B.	178 V	31.57931	072.97683	−	−	−
2	134 J.B.	173 V	31.59705	072.96338	***+***	+	+
3	223 J.B.	169 V	31.62043	072.96727	−	−	−
4	Moza Rajoya	176 R	31.66198	072.97448	***+***	+	+
5	M.Thattian	184 R	31.68778	072.97489	−	−	−
6	M. Johdpur	180 R	31.69026	072.97472	−	−	−
7	Ahmad Nagar	182 R	31.77321	072.89966	−	−	−
8	Ahmad Pur	187 R	31.79090	072.87729	−	−	−
9	M. kanwewala	183 R	31.81763	072.82544	−	−	−
10	Moza Judhi	186 R	31.82471	072.81122	−	−	−
11	202 J.B.	192 V	31.84161	072.83868	−	−	−
12	Kote Qazipur	190 R	31.84774	072.84970	−	−	−
13	Biewal	187 R	31.90920	072.94682	−	−	−
14	Kalowal	182 R	31.90423	072.95624	−	−	−
15	Jand wala	185 R	31.88589	072.99015	−	−	−
16	Pungu Morr	184 R	31.84625	072.96008	***+***	+	+
17	Moza Johdabad	183 R	31.83006	072.93907	−	−	−
18	M.Hussainabad	188 R	31.82157	072.92782	***+***	+	+
19	M.Mohsanabad	178 R	31.79400	072.89456	−	−	−
20	Moza Vaisaan	176 R	31.78678	072.88346	−	−	−
21	241 J.B.	178 V	31.72044	072.95988	−	−	−
22	Moza Bukharian	177 R	31.70576	072.95381	−	−	−
23	Abbas nagar	180 R	31.68935	72.93636	−	−	−
24	Kandiwal	177 R	31.67671	72.90760	−	−	−
25	241 J.B.	179 V	31.66678	72.87928	−	−	−
26	Thatta Jahania	177 R	31.66310	72.86660	***+***	+	+
27	Adil wala bangla	169 V	31.66029	72.85530	−	−	−
28	194 J.B.	171 V	31.65541	72.83943	−	−	−
29	94 J.B.	179 V	31.64864	72.82515	−	−	−
30	202 J.B.	179 V	31.62264	72.77077	***+***	+	+
31	Moza Nitthar	167 R	31.59611	72.70949	−	−	−
32	254 J.B.	168 V	31.58803	72.69275	−	−	−
33	Jamya abad	168 T	31.55409	72.64627	***+***	+	+
34	Gondlan wali	167 R	31.53430	72.66215	−	−	−
35	Chenab mills Ltd	166 C	31.51384	72.67929	−	−	−
36	Bhawana city	170 C	31.50520	72.68622	−	−	−
37	129 J.B.	172 V	31.50039	72.69027	−	−	−
38	41 J.B.	165 V	31.49171	72.69737	−	+	+
39	223 J.B.	169 V	31.45835	72.72484	−	−	+
40	Rahmuana	181 V	31.42409	72.75250	−	−	−

Note: Villages with official code (V), JB, stand for canal irrigation official code. R stands for River Chenab basin areas and C stands for City/Town area. Positive (+) sign indicates presence of RKN; negative (−) indicates absence of RKN in the rice field.

**Table 2.** Prevalence of *M. graminicola* in district Faisalabad.

					Prevalence		
Sr #	Locations	Elevation	Latitude	Longitude	2014	2015	2016
1	217 R.B.	175 V	31.44325	073.00077	−	−	−
2	61 J.B.	176 V	31.45346	072.97887	−	−	−
3	57 J.B.	177 V	31.45328	072.98147	−	−	−
4	58 J.B.	186 V	31.46937	072.99122	***+***	***+***	***+***
5	52 J.B.	181 V	31.49570	073.00120	−	−	−
6	54 J.B.	186 V	31.500394	072.99582	−	−	−
7	51 J.B.	180 V	31.51203	072.98117	−	−	−
8	53 J.B.	180 V	31.52299	072.97618	−	−	−
9	49 J.B.	186 V	31.56101	072.98678	−	−	−
10	218 J.B.	173 V	31.43074	072.97399	***+***	***+***	***+***
11	64 J.B.	173 V	31.39198	072.93964	−	−	−
12	68 J.B.	174 V	31.37310	072.92706	−	−	−
13	66 J.B.	178 V	31.40154	72.97310	−	−	−
14	275 J.B.	169 V	31.34735	72.83235	−	−	−
15	70 J.B.	181 V	31.36304	72.90952	−	***+***	***+***
16	289 R.B.	172 V	31.33934	072.99238	−	−	−
17	245 R.B.	177 V	31.33586	073.01232	−	−	−
18	296 R.B.	179 V	31.33620	073.04997	−	−	−
19	254 R.B.	166 V	31.33576	073.00168	−	−	−
20	269 R.B.	167 V	31.19880	072.99097	−	−	−
21	135 G.B.	169 V	31.14848	072.98298	−	−	−
22	UAF	179 C	31.43735	073.07427	***+***	***+***	***+***
23	Samundari City	168 C	31.05557	072.97504	−	−	−
24	71 G.B.	165 V	31.05584	072.97427	−	−	***+***
25	73 G.B.	165 V	31.05005	072.99210	−	−	−
26	442 G.B.	172 V	31.04441	073.00982	***+***	***+***	***+***
27	393 G.B.	172 V	31.02308	073.07303	−	−	−
28	423 G.B.	178 V	31.07426	073.13823	−	−	−
29	424 G.B.	172 V	31.08084	073.14034	***+***	***+***	***+***
30	430 G.B.	171 V	31.11562	073.15054	−	−	−
31	172 G.B.	178 V	31.14319	073.14831	−	−	−
32	171 G.B.	178 V	31.16456	073.13882	−	−	−
33	39 G.B.	176 V	31.20585	073.17356	***+***	***+***	***+***
34	41 G.B.	182 V	31.20572	073.17418	−	−	−
35	54 G.B.	183 V	31.26873	073.29616	−	−	−
36	21 G.B.	191 V	31.30716	073.38556	−	−	−
37	205 R.B.	186 V	31.43253	073.23050		***+***	***+***
38	66 G.B.	181 V	31.34015	073.33852	***+***	***+***	***+***
39	216 R.B.	183 V	31.38091	073.22599	−	−	−
40	229 R.B.	184 V	31.39439	073.20733	***+***	***+***	***+***

Note: Villages with official code (V), JB, RB and GB stand for canal irrigation official code. C indicates a City/Town area. Positive (+) sign indicates presence of RKN and negative (−) indicates absence of RKN in the rice field.

### Infection and infestation severity of *M. graminicola*

The severity of *M. graminicola* attack on rice roots in infested fields was based on different attributes like galling index, juveniles per root system, juveniles per 100 mL of soil, number of stylet-bearing nematodes (SBN), and the number non-stylet-bearing nematodes (NSBN) from both of the surveyed districts (Tables 3 and 4). The highest galling index was observed in Chiniot and Faisalabad during 2016 and 2014, respectively. *M. graminicola* attack was observed at both districts with significantly high galling index indicative of high infestation of *M. graminicola*. In Chiniot, the highest juvenile/root sample population was recorded in 2016. The highest population of juveniles/soil sample and non-stylet-bearing nematodes was observed in 2014, while the maximum recorded number of stylet-bearing nematodes was from Chiniot in 2015. During 2016, the highest population of juveniles/root sample and stylet-bearing nematodes was observed in Faisalabad, while the highest number of juveniles/soil sample and non-stylet-bearing nematodes was recorded during 2015.

### The occurrence of *M. graminicola* on alternate hosts

The infection of *M. graminicola* was recorded on eleven alternate hosts. All examined alternate hosts showed varying degrees of *M. graminicola* infestation (Table 5). Among them, the maximum juveniles/root sample, juveniles/soil sample, number of stylet-bearing nematodes, and number of non-stylet-bearing nematodes were recovered from *Echinochloa crusgalli*. The lowest number of juveniles/root sample, juveniles/soil sample, and non-stylet-bearing nematodes were observed in *Brassica oleracea*. The lowest number of stylet-bearing nematodes was recorded in *Trigonella foenum-graecum*. Most of the infested fields were canal-irrigated and a few were tube-well irrigated. Both types of irrigation systems generally favor nematode attack, but nematode populations were higher for in-canal irrigation than tube-well. Most of the farmers have a wheat-rice cropping system and only a few of them use a rice-vegetable cropping system (data not shown). In cropping systems that include vegetables, nematode infection was higher on alternate hosts. Grasses like *Cyperus rotundus*, *Dactylocteniuma egyptium*, *Echinochloa crusgalli*, *Eclipta alba*, and *Paspalum distichum* were reported as alternate hosts for rice-vegetable cropping systems, while *Avena fatua*, *Phalaris minor*, and *Rumex dentatus* were recorded as alternate hosts in the rice-wheat cropping system.

### Perineal pattern-based morphological characterization

**Figure 3:**
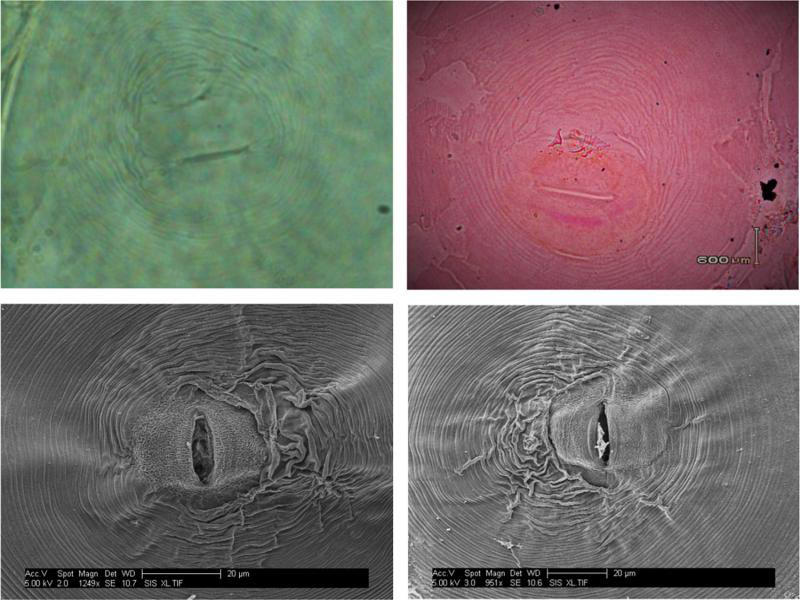
Perineal pattern of *M. graminicola* by light (above) and electron (below) microscopy.

The morphological examination of perineal patterns among obtained isolates showed that they were oval to circular shaped, dorsoventral, moderate in height of the arc, and with no lateral incisures or gaps. The tail tip showed prominent, coarse, and well-separated striae. The obtained perineal patterns were similar to previously described patterns for *M. graminicola* (Figure 3).

### Molecular characterization through ITS amplification and sequencing

**Figure 4:**
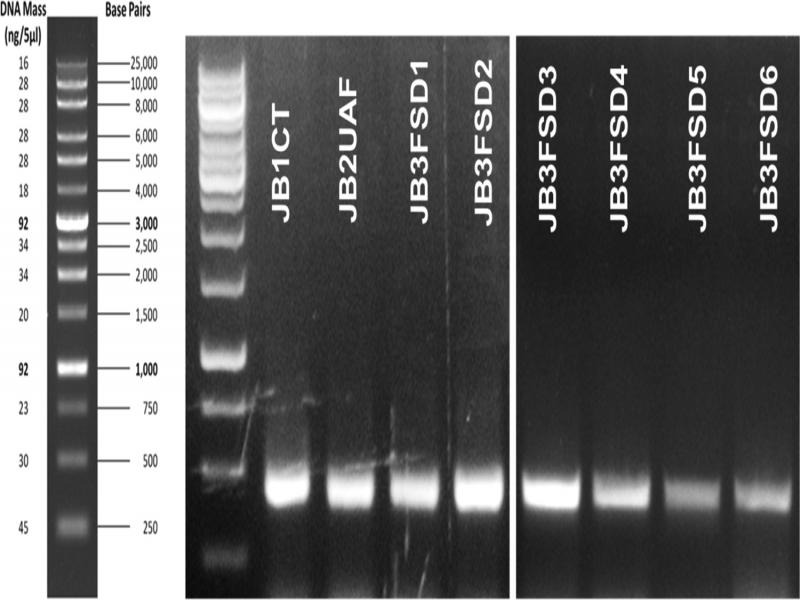
ITS rDNA PCR amplification products using forward primer rDNA2 and reverse primer rDNA1.58 s. Isolate names are given in white text on the upper side of their respective band in the gel; DNA ladder is to the left of the gel.

**Figure 5:**
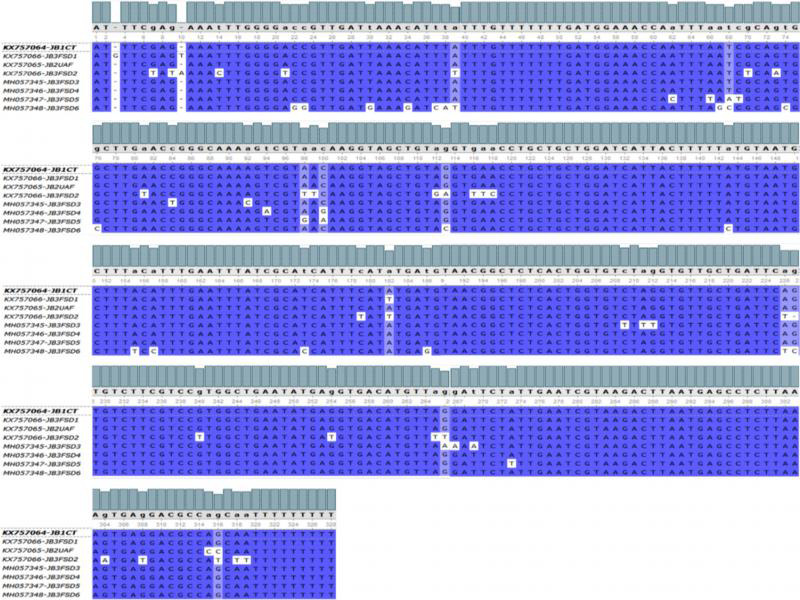
Multiple alignment of ITS sequences from different isolates collected in Faisalabad and Chiniot. The NCBI Genbank accession numbers and isolate names are given in the start of sequences. Complete bars at the top of the sequences show the degree of conservation of different nucleotides in the ITS sequence among different isolates. Similarly, base conservation is also denoted by the capitalized nucleotide alphabet.

PCR amplification and sequencing yielded eight unique sequences. The isolates gave similar-sized bands of 326–328 bp on a gel stained with ethidium bromide (Figure 4). GenBank accession numbers for each of the obtained sequences are given in Tables 3–6. *M. graminicola* isolates JB1CT, JB2 UAF, JB3FSD1, JB3FSD2, JB3FSD3, JB3FSD4, JB3FSD5, and JB3FSD6 (accessions KX757064, 326 bp, KX757065, 328 bp, KX757066, 326 bp, KX757067, 326 bp, MH057345, 326 bp, MH057346, bp, MH057347, bp, and MH057348, 326 bp, respectively) showed 98–100% similarity with GenBank accession numbers KF250491, DQ909030, KF250491, MG773553, KF250481, KJ572383, JF949754, and DQ909040, which were previously curated as *M. graminicola*. The multiple-sequence alignment of the eight isolates demonstrates that there were very few single-nucleotide polymorphisms (SNPs) between the ITS sequences of these isolates (Figure 5). When trimmed for phylogenetic analysis, JB3FSD1 was no longer unique and was removed from subsequent analyses.

**Table 3.** Incidence of *M. graminicola* at different locations of district Chiniot.

Year	Location	Galling index	Juveniles/root sample	Juveniles/soil sample	Stylet bearing nematodes	Non-stylet bearing nematodes
2014	134 J.B	2.20 b	300.80 b	140.40 d	4.20 bc	2.80 e
	Moza Rajoya	3.80 a	348.60 ab	280.80 b	3.80 c	9.40 cd
	Pungu Mor	2.60 ab	290.40 b	108.80 d	1.60 d	11.40 bcd
	Jamya Abad	3.20 ab	390.40 a	119.00 d	3.80 c	12.00 bc
	Jandwala	4.00 a	297.20 b	211.40 c	5.20 ab	8.40 d
	223 JB-2	3.80 a	336.60 ab	347.20 a	5.80 a	14.00 ab
	Rahmuana	3.20 ab	294.00 b	184.00 c	5.40 ab	17.20 a
2015	134 J.B	3.40 a	172.20 a	117.80 f	6.20 cd	7.20 d
	Moza Rajoya	3.20 a	117.40 c	242.60 c	2.80 f	9.20 cd
	Pungu Mor	2.80 a	163.20 ab	136.40 e	7.40 bc	13.00 a
	Jamya Abad	3.20 a	126.40 bc	135.80 e	9.20 a	10.00 bc
	Jandwala	2.80 a	156.80 abc	107.00 g	5.80 de	10.40 bc
	223 JB-2	3.20 a	134.00 abc	194.60 d	4.80 e	9.20 cd
	Rahmuana	3.60 a	130.80 abc	279.60 b	7.80 b	11.80 ab
	202 JB	3.40 a	131.80 abc	292.20 a	7.40 bc	10.40 bc
2016	134 J.B	3.40 ab	278.00 bc	218.00 b	5.20 c	4.00 c
	Moza Rajoya	3.20 ab	268.60 c	298.80 a	9.00 a	10.20 ab
	Pungu Mor	2.80 b	332.80 abc	185.20 e	8.20 ab	7.20 bc
	Jamya Abad	3.00 b	349.40 abc	213.80 bc	7.40 b	9.40 ab
	Jandwala	3.40 ab	380.80 a	199.20 d	5.80 c	8.80 b
	223 JB-2	4.20 a	292.60 bc	172.60 f	5.20 c	9.00 b
	Rahmuana	3.80 ab	280.80 bc	211.40 bc	5.60 c	6.80 bc
	202 JB	3.80 ab	362.00 ab	204.80 cd	5.00 c	8.00 b
	129 JB	2.80 b	330.60 abc	187.20 e	7.20 b	12.60 a

Note: The means followed by the same letters in a column are not significantly different by LSD test (*c* level).

**Table 4.** Incidence of *M. graminicola* at different locations of Faisalabad district.

Year	Location	Galling index	Juveniles/root sample	Juveniles/soil sample	Stylet bearing nematodes	Non-stylet bearing nematodes
2014	58 J.B.	2.60 b	334.80 ab	157.20 e	5.60 bc	9.00 c
	218 R.B.	3.60 ab	352.20 a	131.60 g	4.80 cd	8.80 c
	70 J.B.	1.40 c	340.00 a	219.80 b	4.80 cd	11.00 ab
	442 G.B.	3.60 ab	332.40 ab	147.60 f	4.80 cd	10.60 abc
	39 G.B.	3.00 b	266.20 b	238.00 a	6.00 abc	10.00 bc
	424 G.B.	2.60 b	352.60 a	172.20 d	6.40 ab	9.20 bc
	205 R.B.	3.20 ab	320.00 ab	184.00 c	7.00 a	12.00 a
	UAF	4.20 a	280.80 ab	113.60 h	4.20 d	10.40 abc
2015	58 J.B.	2.60 ab	336.00 a	205.60 b	5.40 cd	9.20 c
	218 R.B.	2.20 b	342.20 a	130.20 e	5.20 cd	8.80 c
	70 J.B.	3.00 ab	331.00 a	137.40 e	4.20 d	9.80 bc
	442 G.B.	2.40 ab	338.40 a	148.80 de	5.20 cd	9.80 bc
	39 G.B.	3.20 ab	276.20 a	185.00 bc	7.80 a	10.00 bc
	424 G.B.	3.80 a	339.60 a	128.60 e	4.80 cd	9.20 c
	205 R.B.	2.20 b	330.60 a	308.40 a	7.00 ab	10.60 abc
	UAF	3.40 ab	292.60 a	127.80 e	5.80 bc	9.40 bc
	209 R.B.	2.80 ab	349.40 a	172.60 cd	8.20 a	11.80 ab
	66 G.B.	2.60 ab	352.80 a	100.40 f	7.20 a	13.00 a
2016	58 J.B.	2.00 d	361.20 a	106.80 g	5.20 de	9.00 bc
	218 R.B.	2.40 cd	340.00 ab	90.60 h	4.80 de	9.60 abc
	70 J.B.	4.00 a	340.00 ab	190.80 b	5.20 de	10.40 abc
	442 G.B.	2.20 cd	328.40 ab	168.00 c	9.00 a	10.20 abc
	39 G.B.	3.20 bc	277.80 b	209.60 a	5.00 de	9.80 abc
	424 G.B.	3.40 ab	394.00 a	158.80 d	7.20 abc	8.80 bc
	205 R.B.	2.40 bcd	362.00 a	150.60 e	4.20 de	8.40 c
	UAF	3.20 abc	380.80 a	207.00 a	6.00 bcd	9.40 abc
	209 R.B.	2.80 bcd	360.40 a	120.20 f	5.40 cde	9.20 abc
	66 G.B.	3.40 ab	368.60 a	162.60 d	7.60 ab	10.80 ab
	71 G.B.	2.60 bcd	358.00 a	123.20 f	3.60 e	11.20 a

Note: The means followed by the same letters in a column are not significantly different by LSD test (*P* = 0.05 level).

**Table 5.** Incidence of *M. graminicola* on different alternate hosts.

Sr #	Local name	Botanical name	Juveniles /root sample	Juveniles/soil sample	Stylet bearing nematodes	Non-stylet bearing nematodes
1	Della	*Cyperus rotundus*	826.6 bc	145.6 b	11.2 de	6.6 b
2	Madhana Grass	*Dactyloctenium aegyptium*	700.8 cd	100.0 de	11.2 de	4.4 cd
3	Barnyard grass	*Echinochloa crusgalli,*	4914.6 a	424.0 a	29.6 a	9.4 a
4	False Daisy	*Eclipta alba*	646.0 d	100.6 de	22.4 b	6.4 bc
5	Knotgrass	*Paspalum distchum*	494.2 e	91.2 e	14.6 cd	5.4 bcd
6	Toothed Dock	*Rumex dentatus*	783.8 cd	121.0 cd	23.0 b	4.6 bcd
7	Jangli gai	*Avena fatua,*	776.2 cd	101.6 de	18.8 bc	5.0 bcd
8	Dumbi Sitti	*Phalaris minor*	942.8 b	129.4 bc	8.4 e	4.0 d
9	Cauliflower	*Brassica oleracea*	142.2 f	43.4 g	10.6 de	3.4 d
10	Coriander	*Coriandrum sativum* L.	143.8 f	85.4 ef	10.2 de	3.6 d
11	Maithi	*Trigonella foenum-graecum* L.	178.6 f	64.4 fg	7.6 e	5.2 bcd

Note: The means followed by the same letters in a column are not significantly different by LS D test (*p* = 0.05 level).

**Table 6.** ITS sequences of *M. graminicola* isolates with GenBank accession numbers.

Location	Elevation	Latitude	Altitude	Isolate	GenBank Accession	Nucleotide
Rajoya	176	31.66198	072.97448	JB1CT	KX757064.1	326 bp
UAF	179	31.43735	073.07427	JB2 UAF	KX757065.1	328 bp
58 J.B	186	31.46937	072.99122	JB3FSD1	KX757066.1	326 bp
218 J.B	173 V	31.43074	072.97399	JB3FSD2	KX757067.1	326 bp
442 G.B	172 V	31.04441	073.00982	JB3FSD3	MH057345.1	326 bp
424 G.B	172 V	31.08084	073.14034	JB3FSD4	MH057346.1	326 bp
205 R.B	186 V	31.43253	073.23050	JB3FSD5	MH057347.1	326 bp
70 J.B	181 V	31.36304	72.909520	JB3FSD6	MH057348.1	326 bp

### Phylogenetic analysis

**Figure 6:**
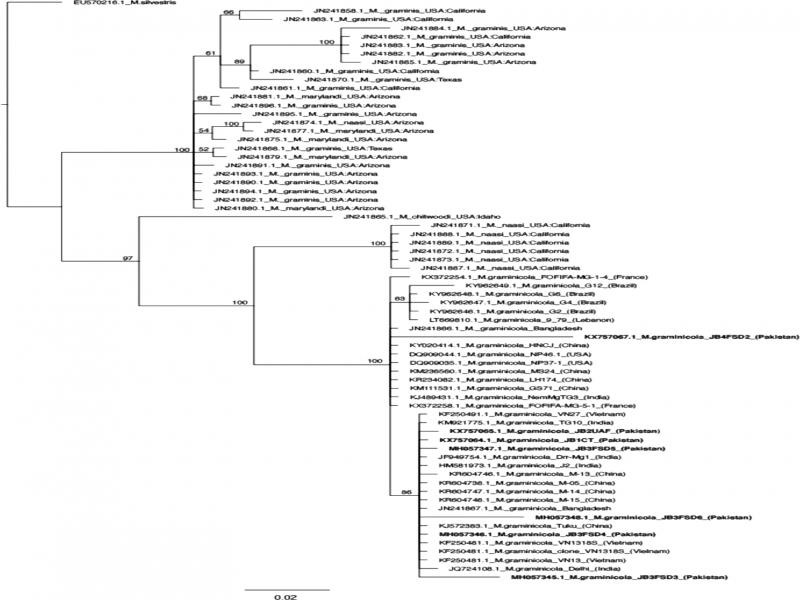
Phylogenetic tree of *M. graminicola* isolates based on ITS sequences. Sequences from this study are in bold. Taxon labels are Genbank accession numbers followed by species epithet, isolate code, and geographic location of the sequenced isolates. Scale bar represents 2% sequence divergence.

The Bayesian solution is presented in Figure 6. Bayesian posterior probabilities, represented as a percentage, are mapped at nodes where support is greater than 50%. The resulting phylogeny shows that six of the isolates sequenced in this study form a monophyletic clade with the other Asian isolates, including those from India, Nepal, China, and Vietnam. Isolate KX757067 belongs to an unresolved polytomy that contains isolates from Asia as well as Europe and South America. The relationships among the isolates are poorly resolved due to lack of synapomorphies. Three isolates from Pakistan, KX757067, MH057348, and MH057345, have several unique nucleotide substitutions, and thus longer branch lengths, relative to the other isolates of *M. graminicola* (Figure 6).

## Discussion

Chiniot and Faisalabad are agriculturally important and are considered some of the most fertile districts in Central Punjab. Rice is also cultivated in these districts with rice-wheat cropping systems. The results of this study reveal variation in the prevalence and infestation rate of *M. graminicola* in Faisalabad and Chiniot rice fields. Several reports have documented the prevalence of *M. graminicola* in rice-cropping systems in different countries (Pokharel et al., 2007; Win et al., 2011; Anil et al., 2011; Pascual et al., 2014). *M. graminicola* has been predominantly reported from lowland production conditions of rice that are common in Chiniot and Faisalabad districts (Pokharel et al., 2007). Most of the rice fields in the present study were infested with *M. graminicola* that is not common in Punjab, Pakistan, because previously *M. incognita* has been reported as the predominant RKN prevailing in agroecosystems of Pakistan, which primarily attacks vegetable crops (Anwar et al., 2012). Faisalabad and Chiniot districts have sandy loam soil, a semiarid environment, and are located in the central region of Punjab, Pakistan. The geographic distribution of RKNs depends on environmental factors such as moisture, soil type, and temperature (Sasser and Triantaphyllou, 1977). Nematode abundance and distribution are directly influenced by soil properties and type of irrigation (Nielsen et al., 2014). Sandy soils generally show higher penetration and development of *Meloidogyne* spp. (Ogbuji, 2004). Soils with a higher percentage of sand had higher abundances of *M. incognita* (Lawrence et al., 1997). Therefore, sandy loam soil of the surveyed regions could be considered important for enhanced development and penetration of *M. graminicola*. RKNs are poikilothermic in nature and require elevated temperatures to increase their rates of development on different cropping systems (Van der Waals et al., 2013). The subtropical climate of Chiniot and Faisalabad districts favors the development of *M. graminicola* in rice-cropping systems.

*Meloidogyne* species have an extensive host range, including grasses, weeds, field, and vegetable crops (Sasser and Freckman, 1987). During our survey, seven alternate hosts of *M. graminicola* were also recorded. Plants were classified as alternate hosts based on prevalence, galling index, RKNs per g root, and RKNs per g soil. Previously, we have reported these plant species as alternate hosts of *M. graminicola* in Pakistan (Jabbar et al., 2016). These alternate hosts help nematodes to persist through summer and winter and act as an important reservoir of nematodes (Queneherve et al., 1995).

Morphological identification based on perineal patterns has been the standard criteria used to identify *Meloidogyne* species since 1949 (Chitwood, 1949). Based on light and scanning electron microscopy, the perineal pattern of isolates from Pakistan is similar to previously described patterns of *M. graminicola* (Yik and Birchfield, 1978; Bernard and Eisenback, 1997) with slight variation, overlapping with the patterns of *M. trifoliophila* and *M. oryzae*. However, perineal patterns alone are insufficient to confirm the identity of *Meloidogyne* spp. as perineal patterns for *M. graminicola*, *M. oryzae*, and *M. trifoliophila* may be conflated (Maas et al., 1978; Pokharel et al., 2007).

To the best of our knowledge, this is the first report of ITS sequences of *M. graminicola* from Pakistan. Sequence similarity analysis was concordant with morphological analyses, and phylogenetic analysis confirmed that our isolates nested well within the other *M. graminicola* sequences available in GenBank, confirming that they are conspecific.

Several studies have demonstrated that ITS sequencing is not only a useful diagnostic approach for *Meloidogyne*, *Globodera*, *Heterodera*, *Longidorus*, *Xiphinema*, and *Pratylenchus* spp. (Powers, 2004), but also for phylogenetic analysis of a number of species in *Heterodera, Meloidogyne*, and *Bursaphelenchus* (Hugall et al., 1999; Powers, 2004). Variability in ITS sequences among the isolates of Pakistan was also observed based on SNPs. The variability in the ITS sequences indicated that amplified ITS regions of *Meloidogyne* species might be useful for population studies. The variability in ITS sequences within an isolate observed in this study could be due to the presence of multiple alleles and/or multiple copies of the sequences that are reported previously in other RKN species (Powers, 2004). Indeed, the high degree of genetic diversity among the Pakistani isolates relative to other regions suggests Pakistan as a possible ancestral area for the Asian isolates of this species (Figure 6).

Our results confirmed that all Pakistani sequenced isolates of root-knot nematode collected from rice were *M. graminicola*. The populations are quite morphologically homogeneous, with only slight variations in morphometric characters and virulence. Our results also confirm the utility of ITS sequences to differentiate *M. graminicola* from other common species of RKNs and reveal considerable variation among Pakistani isolates and relative isolates from other parts of Asia. Our findings confirm the need for further studies on *M. graminicola* biology, genetics, and management.

## Conclusion

The rice-growing fields of Chiniot and Faisalabad, Central Punjab, Pakistan are infested with *M. graminicola*. The subtropical climate, monoculture, high cropping intensity, and sandy loam soils of the areas surveyed likely contribute to the widespread prevalence of *M. graminicola* in Pakistan. *M. graminicola* from our survey displayed infestation on seven alternate hosts in rice-wheat and rice-vegetable cropping systems that provide an alternate means for survival in the absence of agricultural crops. We show that a combination of molecular and morphological traits is a quick and reliable means to accurately identify *M. graminicola*. Phylogenetic analysis and genetic diversity suggests Pakistan as a putative ancestral area. *M. graminicola* is a significant pest of the rice-cropping system of Central Punjab, Pakistan, warranting the adoption of necessary control measures for its management.
